# Gastrointestinal Histoplasmosis Presenting as an Acute Abdomen with Jejunal Perforation

**DOI:** 10.1155/2018/8923972

**Published:** 2018-01-08

**Authors:** Bryan R. Anderson, Jaron Marriott, Chinthaka Bulathsinghala, Humayun Anjum, Salim Surani

**Affiliations:** ^1^Bay Area Medical Center, Corpus Christi, TX, USA; ^2^Texas A&M University, College Station, TX, USA

## Abstract

**Introduction:**

Gastrointestinal histoplasmosis (GH) is a well-described albeit uncommon disease. It is found almost exclusively in the immunocompromised host, especially those with untreated HIV and low CD4 counts. Presentation with intestinal perforation is seen mostly commonly in the colon. We present a patient with jejunal perforation, and there have been only 3 previous cases reported in the literature.

**Case:**

A 39-year-old male with known, untreated HIV presented to the ED with an acute abdomen after experiencing worsening intermittent abdominal pain for 2 months before that was associated with nausea, vomiting, diarrhea, and weight loss. CT of the abdomen and pelvis revealed evidence of gas in the mesentery, small bowel thickening, edema, and free fluid in the abdomen. Emergency exploratory laparotomy was conducted. Intraoperative findings included a perforated jejunum that was studded with nodular lesions as well as mesenteric masses. Histopathologic exam of these mesenteric masses and jejunal lesions were positive for histoplasmosis.

**Conclusion:**

Disseminated histoplasmosis is a life-threatening disease that occurs nearly exclusively in immunocompromised hosts. Untreated, mortality is as high as 80%. This rare presentation with jejunal perforation highlights the need for awareness of histoplasmosis involvement throughout the entirety of the GI tract.

## 1. Introduction

Gastrointestinal histoplasmosis is a well-described phenomenon, occurring almost exclusively in immunocompromised patients. An estimated 70–90% of patients with disseminated histoplasmosis have GI involvement. Of these, only 3–12% are believed to have GI-related symptoms [1]. The most commonly involved sites are the colon and distal ileum, with weight loss, abdominal pain, fever, and diarrhea being the most frequent symptoms. Several case reports and case series have described bowel obstruction and, rarely, perforation, the most common sites being colon or ileum [2–9]. Jejunal perforation has been reported only three times in our literature review [2, 10, 11]. We hereby present an HIV patient presenting with an acute abdomen found to have diffuse jejunal and mesenteric disease. Exploratory laparotomy revealed jejunal perforation.

## 2. Case

A 39-year-old HIV-positive male presented to the Emergency Department complaining of severe, sharp pain diffusely across the abdomen that had awoken him from sleep at 01 : 30 AM. He had been experiencing milder, intermittent abdominal pain for 2 months before. Additionally, he admitted to chills, weight loss, loss of appetite, nausea, and occasional diarrhea and vomiting for 1 month. There was no reported hemoptysis, hematochezia, melena, or fevers. He had a cough with green sputum for 2 months as well.

He had a past medical history significant for HIV due to IV drug use diagnosed 10 years earlier that he had neglected to treat. He has hepatitis C as well. He previously has abused alcohol but had quit 2 months ago, and quit IV drug use 11 years ago. He has less than 10 pack/year smoking. He has been in a monogamous relationship with his girlfriend for multiple years and denies any history of risky sexual behaviors including having polygamous or homozygous relationship. He has lived in southern Texas his whole life, an area of the US known to be mildly endemic with histoplasma species. He had no significant travel history.

He was afebrile with a temperature of 98.2° Fahrenheit, stable blood pressure, tachycardia, and tachypnea with a heart rate of 110/bpm and respiratory rate of 22/min. Physical exam revealed a malnourished man in distress due to abdominal pain. On abdominal exam, he was very tender with diffuse guarding and rigidity in all quadrants. Initial lab work revealed white blood cell count of 2.95/10^3^/*μ*L. Absolute CD4 count was 26, and viral load was 162,000. His remaining initial lab work (with normal values) was as follows: WBC 2.95 × 10^3^/microliter (4.8–10.8 × 10^3^), absolute neutrophil count 2.57 × 10^3^/microliter (1.8–7.7 × 10^3^), absolute lymphocyte 0.20 × 10^3^/microliter (1.0–4.8 × 10^3^), absolute monocytes 0.18 × 10^3^/microliter (0.0–0.8 × 10^3^), eosinophils 0.00 × 10^3^/microliter, and basophils 0.00 × 10^3^/microliter. Hemoglobin was 11.4 g/dl (12.0–16 g/dl), hematocrit was 35.4%, and platelet count was 248 × 10^3^/microliter (150–450 × 10^3^/microliter).

**Figure 1:**
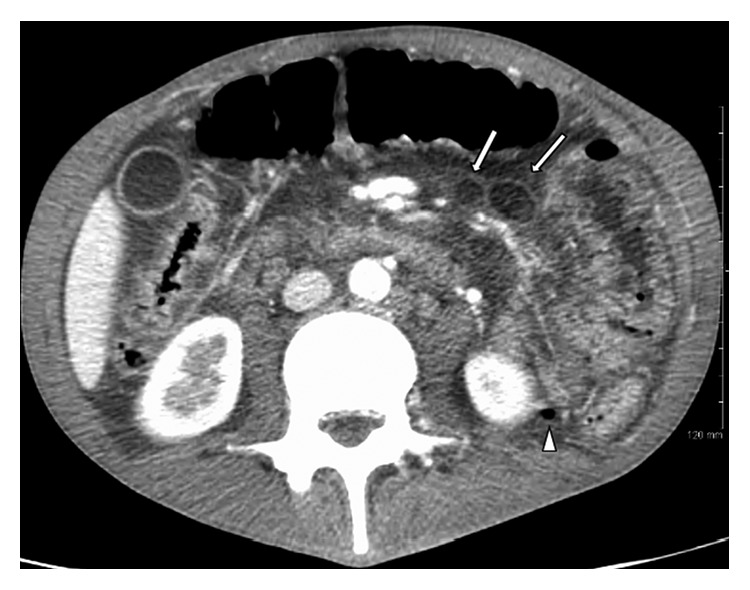
CT abdomen/pelvis with IV contrast (axial). White arrows: mesenteric masses found to be histoplasmosis after surgical resection. White arrowhead: free air in the mesentery from jejunal perforation.

**Figure 2:**
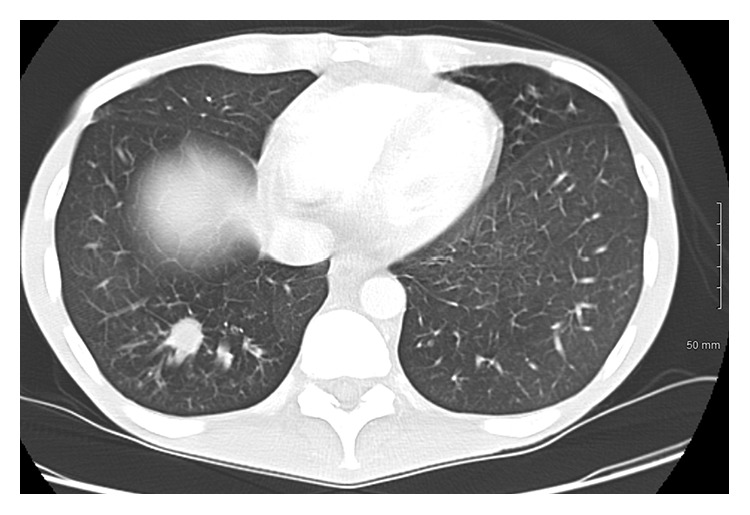
Contrasted CT of the chest showing a 16 mm right lower lobe nodule.

CT of the abdomen and pelvis (Figure 1) revealed gas bubbles scattered throughout the left mesentery, multiple areas of small bowel thickening and edema, evidence of free fluid in the abdomen, and mesenteric masses (Figure 1). These findings supported bowel perforation. Additionally, workup included CT angiogram (CTA) of the chest, to exclude the pulmonary embolism. The CTA showed 16 mm right lower lobe nodule (Figure 2), as well as a 6 mm left upper lobe nodule. No hilar or mediastinal adenopathy was seen.

An exploratory laparotomy was performed. This revealed numerous nodular implants throughout the small bowel as well as numerous masses in the mesentery. There was an obvious perforation in the mid to proximal jejunum associated with a nodular lesion that had caused a stricture. The perforated segment was resected along with a representative mesenteric mass. Primary anastomosis was performed noting that the extent of disease involvement in the small bowel prohibited complete resection of all lesions.

**Figure 3:**
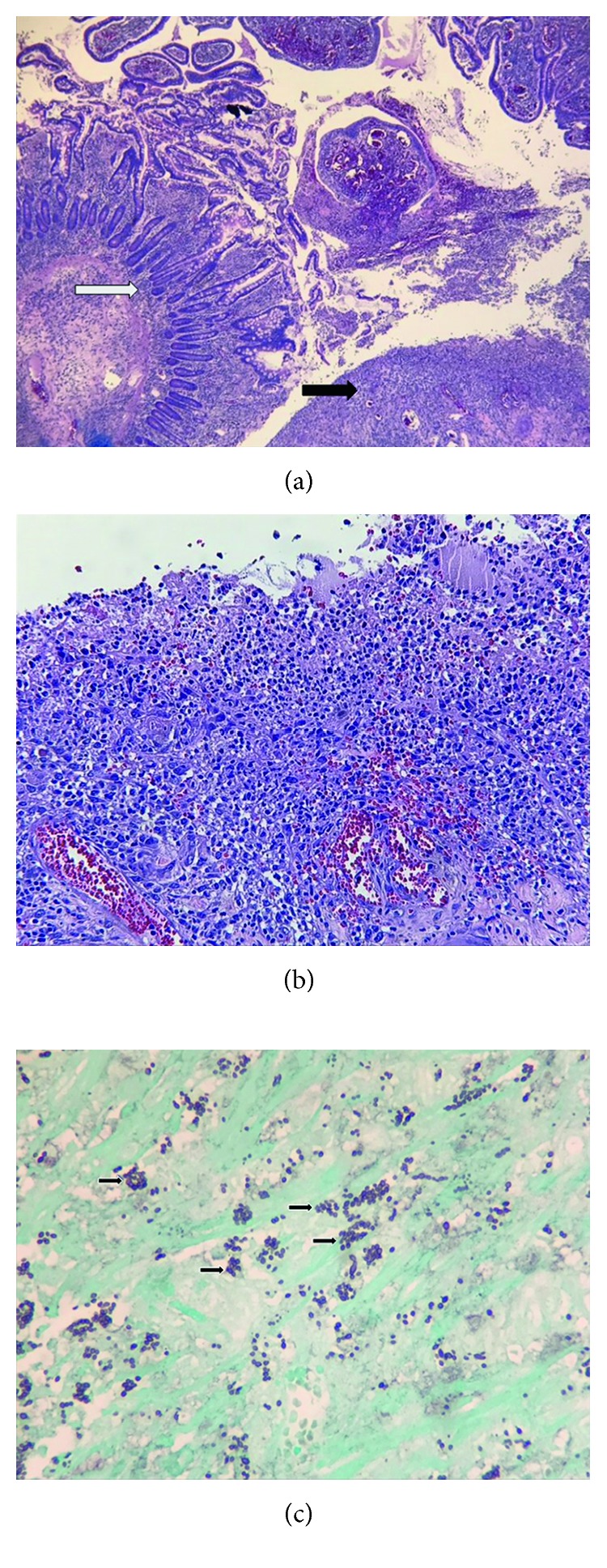
(a) Low power H and E stain showing normal small bowel (white arrow) compared with ulcerated small bowel (black arrow). (b) Higher power H and E stain showing small bowel necrosis and inflammation. (c) GMS silver stain demonstrating numerous histoplasmosis (black arrows).

Histological examination of surgical specimens showed necrotic tissue with yeast consistent with histoplasmosis (Figures 3–3). Urine histoplasma antigen was markedly positive at >25 ng/ml (0.00–0.49).

In regard to his pulmonary nodule seen on the CTA, the patient underwent bronchoscopy. Minimal diffuse inflammatory changes and thick whitish secretions were seen in the right lower lobe and right upper lobe. Bronchoalveolar lavage (BAL) was performed from left upper and right lower lobe. Gram stain of the BAL fluid showed 3 + WBCs with rare Gram negative rods, 1+ Gram positive cocci, and few coryneform Gram positive rods which was felt to be consistent with normal upper respiratory flora. Cultures grew rapid growing candida species. Pathological examination of the BAL fluid revealed inflammatory debries with yeast forms seen on the silver stain. No biopsies were performed.

The patient was started on oral itraconazole, 200 mg orally TID for 3 days, and then changed to 200 mg orally BID anticipating 12 months of treatment. In addition to prophylactic, trimethoprim/sulfamethoxazole 800 mg/160 mg one tablet orally and azithromycin 1500 mg orally once per week were started. He was not immediately started on antiretroviral therapy due to lack of inpatient access to these medications.

His hospitalization was complicated by high output drainage from his surgical JP drain. This eventually decreased to a more manageable level, and the patient was discharged home with his JP drain in place on day 17 of hospitalization. On discharge, he was referred to a local clinic for treatment of AIDS. He failed to follow-up there, citing financial hardship.

The patient returned to the hospital 1 week after discharge with fever, chills, constipation, and abdominal discomfort with partial small bowel obstruction, which was managed conservatively and good resolution was achieved. Repeat bronchoscopy with BAL was negative except for growing yeast in the BAL. Patient was changed to liposomal amphotericin B intravenously with good clinical response. After 2 weeks of intravenous liposomal amphotericin B, he was switched back to itraconazole 200 mg orally BID, anticipating 12 months of therapy with close follow-up. He was discharged home with an instruction to follow-up at the HIV clinic to be started on antiretroviral therapy and follow-up CT scan of chest in 4 weeks.

## 3. Discussion

*Histoplasma capsulatum* is a dimorphic fungus endemic to the Midwestern and Southeastern United States as well as Central and South America. It is found most readily in soil contaminated with bird or bat guano [12]. It is the most frequent cause of fungal respiratory infections and usually has a self-limited course in immunocompetent hosts. It may also present as a progressive disseminated disease that can be life threatening, especially in the elderly and immunocompromised hosts. HIV-positive individuals with CD4+ cell counts lower than 200 cells/mL are at elevated risk for disseminated histoplasmosis (DH).

Gastrointestinal histoplasmosis (GIH) is uncommon, manifesting symptomatically in 3–12% of patients with DH. Much higher rates of GI histoplasmosis have been reported on autopsy review indicating the presence of disease which is asymptomatic [11]. Nevertheless, it is a rare disease and presents almost exclusively in the immunocompromised host. The most common sites of involvement are the colon and ileum. It becomes less common more proximally in the small intestine [2, 11, 13].

The most common symptoms in those with GIH are weight loss, abdominal pain, fever, and diarrhea. The disease manifests variably as intestinal ulcers, bleeding, strictures, and, rarely, perforation. Several published cases and case series report ileal and colonic perforation. In 2000, Gumbs et al. reported what they believed to be the first report of jejunal perforation [10]. A 2006 case series by Assi et al. report 18 new cases of GI histoplasmosis. Among these, 1 experienced jejunal perforation [13].

## 4. Conclusion

This rare presentation of a jejunal perforation due to disseminated histoplasmosis highlights the need for heightened awareness of this disorder in immunocompromised individuals. The majority of patients of GIH are AIDS patients with low CD4 counts who have not received therapy. The mortality in these patients without therapy is high, approaching 80%. With antifungal therapy, mortality improves to 30%. Our patient was initially treated with itraconazole, but was later changed to liposomal amphotericin B when his pulmonary lesions advanced. With this regimen, he has remained stable, although noncompliance with antiretroviral therapy remains an issue.
